# 2-Benzoylmethyl-4-[(2-benzylidene­ethylidene)amino]-5-(2-thienylmethyl)-2*H*-1,2,4-triazol-3(4*H*)-one

**DOI:** 10.1107/S1600536809012719

**Published:** 2009-04-08

**Authors:** Reşat Ustabaş, Yasemin Ünver, Nevin Suleymanoğlu, Ufuk Çoruh, Kemal Sancak

**Affiliations:** a Department of Middle Education, Faculty of Education, Ondokuz Mayıs University, 55200-Atakum, Samsun, Turkey; b Department of Chemistry, Faculty of Arts and Sciences, Karadeniz Teknik University, 61080-Trabzon, Turkey; c Department of Elementary Education, Faculty of Education, Ondokuz Mayıs University, 55200-Atakum, Samsun, Turkey; d Department of Computer Education and Instructional Technology, Faculty of Education, Ondokuz Mayıs University, 55200-Atakum-Samsun, Turkey

## Abstract

In the mol­ecule of the title compound, C_24_H_20_N_4_O_2_S, the dihedral angle between the triazole and thio­phene rings is 66.80 (4)° and the dihedral angle between the two benzene rings is 63.37 (4)°. An intra­molecular C—H⋯O inter­action results in the formation of a six-membered ring. A π⋯π contact between the benzene rings, [centroid–centroid distance = 3.918 (2) Å] may stabilize the structure. Weak C—H⋯π inter­actions are also present. The S, C and H atoms of the thiophene ring are disordered over two positions and were refined with occupancies of 0.654 (3) and 0.346 (3).

## Related literature

For general background to 1,2,4-triazoles, see: Clemons *et al.* (2004 ▶); Colanceska-Ragenovic *et al.* (2001 ▶); Goss & Strasser-Weippl (2004 ▶); Santen (2003 ▶); Tsukuda *et al.* (1998 ▶); Ünver *et al.* (2008 ▶); Zhu *et al.* (2000 ▶). For related structucres, see: Çoruh *et al.* (2003 ▶); Ünver *et al.* (2006 ▶); Yılmaz *et al.* (2006 ▶); Vrábel *et al.* (2005 ▶). For bond-length data, see: Allen *et al.* (1987 ▶).
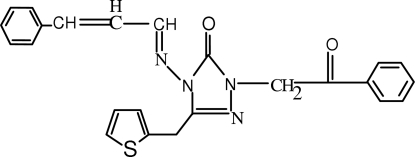


## Experimental

### 

#### Crystal data

C_24_H_20_N_4_O_2_S
*M* *_r_* = 428.50
Monoclinic, 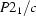

*a* = 17.4972 (3) Å
*b* = 14.7609 (3) Å
*c* = 8.2724 (1) Å
β = 96.395 (1)°
*V* = 2123.25 (6) Å^3^
*Z* = 4
Cu *K*α radiation
μ = 1.59 mm^−1^
*T* = 294 K
0.30 × 0.11 × 0.07 mm

#### Data collection

Bruker CCD 6000 area-detector diffractometer
Absorption correction: none
10706 measured reflections
3697 independent reflections
2561 reflections with *I* > 2σ(*I*)
*R*_int_ = 0.039

#### Refinement

*R*[*F* ^2^ > 2σ(*F* ^2^)] = 0.052
*wR*(*F* ^2^) = 0.175
*S* = 1.06
3697 reflections
314 parameters
H-atom parameters constrained
Δρ_max_ = 0.40 e Å^−3^
Δρ_min_ = −0.22 e Å^−3^

### 

Data collection: *SMART* (Bruker, 1997 ▶); cell refinement: *SAINT* (Bruker, 1997 ▶); data reduction: *SAINT*; program(s) used to solve structure: *SHELXS97* (Sheldrick, 2008 ▶); program(s) used to refine structure: *SHELXL97* (Sheldrick, 2008 ▶); molecular graphics: *ORTEP-3 for Windows* (Farrugia, 1997 ▶); software used to prepare material for publication: *WinGX* publication routines (Farrugia, 1999 ▶).

## Supplementary Material

Crystal structure: contains datablocks global, I. DOI: 10.1107/S1600536809012719/hk2655sup1.cif

Structure factors: contains datablocks I. DOI: 10.1107/S1600536809012719/hk2655Isup2.hkl

Additional supplementary materials:  crystallographic information; 3D view; checkCIF report

## Figures and Tables

**Table 1:** Hydrogen-bond geometry (Å, °)

*D*—H⋯*A*	*D*—H	H⋯*A*	*D*⋯*A*	*D*—H⋯*A*
C42—H42⋯O1	0.93	2.23	2.916 (3)	130
C14—H14⋯*Cg*1^i^	0.93	2.91	3.501 (3)	122
C11—H11*B*⋯*Cg*2^ii^	0.97	2.89	3.816 (3)	159
C48—H48⋯*Cg*3^iii^	0.93	2.79	3.557 (3)	141

Symmetry codes: (i) 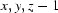; (ii) 
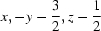
; (iii) 
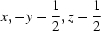
. *Cg*1, *Cg*2 and *Cg*3 are the centroids of the N1/N2/N4/C3/C5, C13–C18 and S1/C32–C35 rings, respectively.

**Fig. 1.**
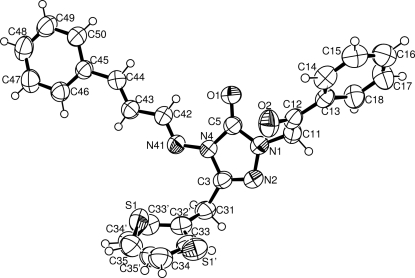
The molecular structure of the title molecule, with the atom-numbering scheme. Displacement ellipsoids are drawn at the 50% probability level.

## supplementary crystallographic information

### Comment

The 1,2,4-triazole compounds posses important pharmacological activities such as antifungal and antiviral activities. Examples of such compounds bearing the 1,2,4- triazole residues are fluconazole, the powerful azole antifungal agent as well as the potent antiviral N- nucleoside ribavirin (Tsukuda *et al.*, 1998; Ünver *et al.*, 2008). The 1,2,4-triazole nucleus is associated with diverse pharmacological activities such as antibacterial, hypoglycemic, antihypertensive, analgesic and specific magnetic properties (Colanceska-Ragenovic *et al.*, 2001; Zhu *et al.*, 2000). It was reported that compounds having triazole moieties such as Vorozole, Anastrozole and Letrozole appear to be very effective aromatase inhibitors very useful for preventing breast cancer (Goss & Strasser-Weippl, 2004; Santen, 2003). There are antimicrobial agents having different structures are frequently used in treatment of microbial infections. However, there is an increasing resistance to these drugs. Moreover, some of azole derivatives used as common antibiotics, such as Amphotericin B posses a toxic effect on humans as well as their antimicrobial effects (Clemons *et al.*,  2004). We report herein the crystal structure of the title compound.

In the molecule of the title compound (Fig 1), the bond lengths (Allen *et al.*,  1987) and angles are within normal ranges. In the triazole ring, N2-C3 [1.292 (3) Å] bond is shorter than N4-C3 [1.373 (3) Å] bond and they are in agreement with the corresponding values in similar structures containing triazole ring, such as [1.290 (3) and 1.384 Å; Yılmaz *et al.*, 2006] and [1.278 (3) and 1.379 (3) Å;Çoruh *et al.*,  2003]. S1-C32 [1.698 (3) Å] and S1-C35 [1.735 (6) Å] bonds also agree with the corresponding values [1.706 (2) and 1.723 (2) Å;Ünver *et al.*,  2006] and [1.676 (4) and 1.689 (3) Å (Vrábel *et al.*,  2005]. Rings A (N1/N2/N4/C3/C5), B (C13-C18), C (S1/C32-C35) and D (C45-C50), are, of course, planar and they are oriented at dihedral angles of A/B = 63.76 (3), A/C = 66.80 (4), A/D = 4.81 (3), B/C = 68.37 (3), B/D = 63.37 (4) and C/D = 63.57 (3) °. Intramolecular C-H···O interaction results in the formation of a six-membered ring having envelope conformation, with atom O1 displaced by 0.300 (3) Å from the plane of the other ring atoms.

In the crystal structure, the π···π contact between the benzene rings, Cg2—Cg2^i^ [symmetry code: (i) -x, -y, -1 - z, where Cg2 is centroid of the ring B (C13-C18)] may stabilize the structure, with centroid-centroid  distance of 3.918 (2) Å. There also exist weak C—H···π interactions (Table 1).

### Experimental

For the preparation of the title compound, 4-[(3-phenyl-allidenamino)-5-thiophen -2-yl-methyl-2,4-dihydro-1,2,4]triazol-3-one (0.01 mol) was refluxed with an equivalent amount of natrium in absolute ethanol (100 ml) for 1 h. Then, ethyl bromoacetophenon (0.01 mol) was added and refluxed for an additional 5 h. The precipitate was filtered off, washed with water and recrystallized from ethanol/water (1:2) (yield; 70.79%, m.p. 433-434 K).

### Refinement

The S, C and H atoms of the thiophene ring were disordered. During the refinement process, the disordered atoms were refined with occupancies of 0.654 (3) and 0.346 (3). H atoms were positioned geometrically, with C-H = 0.93 and 0.97 Å for aromatic and methylene H, respectively, and constrained to ride on their parent atoms, with U_iso_(H) = 1.2U_eq_(C).

### Crystal data

**Table:**

C_24_H_20_N_4_O_2_S	*F*(000) = 896
*M**_r_* = 428.50	*D*_x_ = 1.340 Mg m^−^^3^
Monoclinic, *P*2_1_/*c*	Cu *K*α radiation, λ = 1.54178 Å
Hall symbol: -P 2ybc	Cell parameters from 2561 reflections
*a* = 17.4972 (3) Å	θ = 2.5–68.2°
*b* = 14.7609 (3) Å	µ = 1.59 mm^−^^1^
*c* = 8.2724 (1) Å	*T* = 294 K
β = 96.395 (1)°	Prism, colorless
*V* = 2123.25 (6) Å^3^	0.30 × 0.11 × 0.07 mm
*Z* = 4	

### Data collection

**Table:**

Bruker CCD 6000 area-detector diffractometer	2561 reflections with *I* > 2σ(*I*)
Radiation source: fine-focus sealed tube	*R*_int_ = 0.039
graphite	θ_max_ = 68.2°, θ_min_ = 2.5°
φ and ω scans	*h* = −20→18
10706 measured reflections	*k* = −16→16
3697 independent reflections	*l* = −9→9

### Refinement

**Table:**

Refinement on *F*^2^	Secondary atom site location: difference Fourier map
Least-squares matrix: full	Hydrogen site location: inferred from neighbouring sites
*R*[*F*^2^ > 2σ(*F*^2^)] = 0.052	H-atom parameters constrained
*wR*(*F*^2^) = 0.175	*w* = 1/[σ^2^(*F*_o_^2^) + (0.1091*P*)^2^] where *P* = (*F*_o_^2^ + 2*F*_c_^2^)/3
*S* = 1.06	(Δ/σ)_max_ < 0.001
3697 reflections	Δρ_max_ = 0.40 e Å^−^^3^
314 parameters	Δρ_min_ = −0.22 e Å^−^^3^
Primary atom site location: structure-invariant direct methods	

### Special details

**Table:**

Geometry. All e.s.d.'s (except the e.s.d. in the dihedral angle between two l.s. planes) are estimated using the full covariance matrix. The cell e.s.d.'s are taken into account individually in the estimation of e.s.d.'s in distances, angles and torsion angles; correlations between e.s.d.'s in cell parameters are only used when they are defined by crystal symmetry. An approximate (isotropic) treatment of cell e.s.d.'s is used for estimating e.s.d.'s involving l.s. planes.
Refinement. Refinement of *F*^2^ against ALL reflections. The weighted *R*-factor *wR* and goodness of fit *S* are based on *F*^2^, conventional *R*-factors *R* are based on *F*, with *F* set to zero for negative *F*^2^. The threshold expression of *F*^2^ > σ(*F*^2^) is used only for calculating *R*-factors(gt) *etc*. and is not relevant to the choice of reflections for refinement. *R*-factors based on *F*^2^ are statistically about twice as large as those based on *F*, and *R*- factors based on ALL data will be even larger.

### Fractional atomic coordinates and isotropic or equivalent isotropic displacement parameters (Å^2^)

**Table:**

	*x*	*y*	*z*	*U*_iso_*/*U*_eq_	Occ. (<1)
S1	0.58098 (11)	0.41692 (13)	0.0657 (3)	0.0861 (6)	0.654 (3)
S1'	0.6146 (4)	0.2606 (4)	0.2561 (8)	0.0857 (11)	0.346 (3)
O1	0.88146 (9)	0.54521 (11)	0.47227 (19)	0.0690 (5)	
O2	0.83595 (10)	0.40286 (12)	0.7644 (2)	0.0831 (5)	
N1	0.86815 (11)	0.38928 (12)	0.4521 (2)	0.0637 (5)	
N2	0.82649 (11)	0.32716 (12)	0.3517 (2)	0.0649 (5)	
N4	0.79359 (10)	0.46712 (11)	0.2811 (2)	0.0535 (4)	
N41	0.75222 (11)	0.53233 (12)	0.1882 (2)	0.0599 (5)	
C3	0.78325 (13)	0.37638 (15)	0.2497 (3)	0.0580 (6)	
C5	0.85161 (13)	0.47661 (15)	0.4121 (3)	0.0564 (5)	
C11	0.92508 (14)	0.36161 (16)	0.5806 (3)	0.0660 (6)	
H11A	0.9728	0.3932	0.5693	0.079*	
H11B	0.9345	0.2972	0.5705	0.079*	
C12	0.90121 (14)	0.38067 (14)	0.7490 (3)	0.0598 (6)	
C13	0.96094 (13)	0.37260 (13)	0.8891 (3)	0.0559 (5)	
C14	0.94621 (16)	0.40534 (15)	1.0407 (3)	0.0672 (7)	
H14	0.8981	0.4297	1.0527	0.081*	
C15	1.00083 (17)	0.40240 (17)	1.1714 (3)	0.0744 (7)	
H15	0.9899	0.4253	1.2710	0.089*	
C16	1.07174 (17)	0.36594 (18)	1.1572 (3)	0.0775 (7)	
H16	1.1090	0.3639	1.2465	0.093*	
C17	1.08709 (17)	0.3324 (2)	1.0088 (3)	0.0839 (8)	
H17	1.1348	0.3066	0.9984	0.101*	
C18	1.03279 (15)	0.33661 (16)	0.8763 (3)	0.0727 (7)	
H18	1.0445	0.3149	0.7765	0.087*	
C31	0.72983 (14)	0.34018 (16)	0.1113 (3)	0.0687 (6)	
H31A	0.7351	0.3766	0.0155	0.082*	
H31B	0.7453	0.2789	0.0882	0.082*	
C32	0.64725 (15)	0.33916 (15)	0.1403 (3)	0.07042 (8)	0.654 (3)
C32'	0.64725 (15)	0.33916 (15)	0.1403 (3)	0.07042 (8)	0.346 (3)
C33	0.6141 (7)	0.2793 (8)	0.2307 (16)	0.0822 (19)	0.654 (3)
H33	0.6421	0.2322	0.2832	0.099*	0.654 (3)
C33'	0.5934 (8)	0.3931 (9)	0.070 (2)	0.086 (2)	0.346 (3)
H33'	0.6037	0.4378	−0.0036	0.103*	0.346 (3)
C34	0.5342 (4)	0.2905 (7)	0.2437 (14)	0.0834 (15)	0.654 (3)
H34	0.5044	0.2528	0.3015	0.100*	0.654 (3)
C34'	0.5184 (7)	0.3780 (12)	0.116 (2)	0.086 (2)	0.346 (3)
H34'	0.4743	0.4109	0.0809	0.103*	0.346 (3)
C35	0.5090 (4)	0.3640 (6)	0.1591 (9)	0.0847 (17)	0.654 (3)
H35	0.4585	0.3844	0.1512	0.102*	0.654 (3)
C35'	0.5228 (7)	0.3073 (13)	0.219 (3)	0.082 (2)	0.346 (3)
H35'	0.4807	0.2855	0.2670	0.099*	0.346 (3)
C42	0.75715 (13)	0.61382 (15)	0.2406 (3)	0.0598 (6)	
H42	0.7884	0.6271	0.3361	0.072*	
C43	0.71480 (13)	0.68498 (15)	0.1530 (3)	0.0605 (6)	
H43	0.6822	0.6705	0.0600	0.073*	
C44	0.72076 (13)	0.77056 (15)	0.2004 (3)	0.0637 (6)	
H44	0.7551	0.7813	0.2925	0.076*	
C45	0.68128 (13)	0.85043 (15)	0.1292 (3)	0.0586 (6)	
C46	0.62026 (14)	0.84499 (17)	0.0066 (3)	0.0675 (6)	
H46	0.6026	0.7886	−0.0312	0.081*	
C47	0.58586 (16)	0.9221 (2)	−0.0589 (4)	0.0826 (8)	
H47	0.5453	0.9176	−0.1412	0.099*	
C48	0.61094 (17)	1.0064 (2)	−0.0037 (4)	0.0878 (8)	
H48	0.5874	1.0585	−0.0487	0.105*	
C49	0.67029 (17)	1.01301 (18)	0.1166 (4)	0.0893 (8)	
H49	0.6873	1.0697	0.1542	0.107*	
C50	0.70537 (16)	0.93594 (17)	0.1831 (3)	0.0765 (7)	
H50	0.7459	0.9413	0.2654	0.092*	

### Atomic displacement parameters (Å^2^)

**Table:**

	*U*^11^	*U*^22^	*U*^33^	*U*^12^	*U*^13^	*U*^23^
S1	0.0805 (10)	0.0722 (11)	0.1040 (9)	0.0136 (6)	0.0029 (7)	0.0105 (8)
S1'	0.100 (2)	0.075 (2)	0.0847 (19)	−0.0139 (15)	0.0221 (15)	0.0089 (14)
O1	0.0746 (11)	0.0566 (10)	0.0731 (10)	−0.0050 (7)	−0.0033 (8)	−0.0049 (8)
O2	0.0659 (12)	0.0931 (13)	0.0922 (12)	0.0176 (9)	0.0176 (10)	0.0168 (9)
N1	0.0714 (13)	0.0495 (11)	0.0679 (11)	0.0034 (9)	−0.0022 (10)	−0.0017 (9)
N2	0.0706 (14)	0.0505 (11)	0.0725 (12)	0.0013 (9)	0.0039 (10)	−0.0048 (9)
N4	0.0555 (11)	0.0472 (11)	0.0577 (10)	0.0027 (7)	0.0052 (8)	−0.0001 (8)
N41	0.0625 (12)	0.0545 (12)	0.0623 (10)	0.0014 (8)	0.0055 (9)	0.0053 (9)
C3	0.0605 (14)	0.0507 (13)	0.0638 (13)	0.0001 (10)	0.0114 (11)	−0.0072 (10)
C5	0.0569 (14)	0.0537 (14)	0.0589 (12)	0.0003 (10)	0.0074 (11)	−0.0005 (10)
C11	0.0672 (16)	0.0619 (14)	0.0677 (14)	0.0116 (11)	0.0020 (12)	0.0019 (11)
C12	0.0634 (16)	0.0448 (12)	0.0722 (14)	0.0048 (10)	0.0118 (12)	0.0090 (10)
C13	0.0617 (14)	0.0437 (12)	0.0635 (13)	−0.0012 (9)	0.0127 (11)	0.0058 (9)
C14	0.0737 (17)	0.0634 (15)	0.0688 (15)	0.0020 (11)	0.0277 (14)	0.0052 (11)
C15	0.091 (2)	0.0772 (17)	0.0579 (14)	−0.0105 (14)	0.0212 (15)	0.0020 (12)
C16	0.085 (2)	0.0819 (18)	0.0649 (15)	−0.0121 (14)	0.0065 (14)	0.0073 (13)
C17	0.0701 (18)	0.111 (2)	0.0691 (16)	0.0171 (15)	0.0029 (14)	0.0011 (14)
C18	0.0739 (18)	0.0830 (18)	0.0619 (14)	0.0169 (13)	0.0112 (13)	−0.0054 (12)
C31	0.0737 (16)	0.0609 (14)	0.0715 (14)	−0.0004 (11)	0.0082 (12)	−0.0152 (11)
C32	0.078	0.059	0.073	−0.0035 (11)	0.0036 (11)	−0.0121 (11)
C32'	0.078	0.059	0.073	−0.0035 (11)	0.0036 (11)	−0.0121 (11)
C33	0.090 (3)	0.067 (4)	0.090 (4)	−0.020 (3)	0.012 (3)	0.005 (3)
C33'	0.085 (4)	0.072 (5)	0.099 (4)	−0.001 (4)	0.003 (4)	−0.006 (4)
C34	0.077 (3)	0.076 (4)	0.097 (4)	−0.012 (2)	0.011 (3)	−0.003 (2)
C34'	0.075 (4)	0.078 (4)	0.103 (5)	−0.005 (4)	−0.004 (4)	0.001 (4)
C35	0.069 (3)	0.082 (4)	0.103 (5)	−0.005 (2)	0.009 (3)	−0.006 (3)
C35'	0.069 (4)	0.079 (5)	0.099 (5)	−0.013 (4)	0.007 (4)	−0.009 (4)
C42	0.0619 (15)	0.0551 (14)	0.0624 (13)	0.0041 (10)	0.0067 (11)	0.0016 (10)
C43	0.0617 (15)	0.0560 (14)	0.0632 (12)	−0.0002 (10)	0.0046 (11)	0.0050 (10)
C44	0.0614 (15)	0.0612 (15)	0.0671 (13)	0.0018 (10)	0.0015 (11)	0.0009 (11)
C45	0.0520 (14)	0.0540 (13)	0.0713 (14)	0.0007 (9)	0.0125 (12)	0.0025 (10)
C46	0.0611 (16)	0.0624 (15)	0.0795 (16)	0.0009 (11)	0.0097 (13)	0.0041 (12)
C47	0.0709 (18)	0.084 (2)	0.0921 (18)	0.0095 (14)	0.0054 (15)	0.0170 (15)
C48	0.077 (2)	0.071 (2)	0.118 (2)	0.0145 (14)	0.0189 (18)	0.0249 (17)
C49	0.080 (2)	0.0561 (16)	0.132 (2)	0.0019 (13)	0.0142 (19)	0.0025 (16)
C50	0.0697 (17)	0.0586 (16)	0.0998 (19)	0.0024 (12)	0.0033 (14)	−0.0030 (13)

### Geometric parameters (Å, °)

**Table:**

O1—C5	1.220 (2)	C32—C33	1.331 (9)
N1—C11	1.433 (3)	C33—C34	1.424 (13)
N2—N1	1.388 (2)	C33—H33	0.9300
N4—N41	1.385 (2)	C33'—C34'	1.424 (15)
N4—C3	1.373 (3)	C33'—H33'	0.9300
N4—C5	1.407 (3)	C34—C35	1.339 (6)
N41—C42	1.278 (3)	C34—H34	0.9300
C3—N2	1.292 (3)	C34'—C35'	1.345 (9)
C5—N1	1.354 (3)	C34'—H34'	0.9300
C11—H11A	0.9700	C35—S1	1.735 (6)
C11—H11B	0.9700	C35—H35	0.9300
C12—O2	1.208 (3)	C35'—S1'	1.744 (10)
C12—C11	1.524 (3)	C35'—H35'	0.9300
C12—C13	1.476 (3)	C42—C43	1.435 (3)
C13—C14	1.394 (3)	C42—H42	0.9300
C13—C18	1.380 (3)	C43—C44	1.323 (3)
C14—C15	1.362 (4)	C43—H43	0.9300
C14—H14	0.9300	C44—H44	0.9300
C15—C16	1.370 (4)	C45—C50	1.388 (3)
C15—H15	0.9300	C45—C46	1.391 (3)
C16—C17	1.378 (3)	C45—C44	1.457 (3)
C16—H16	0.9300	C46—C47	1.371 (3)
C17—H17	0.9300	C46—H46	0.9300
C18—C17	1.369 (4)	C47—C48	1.380 (4)
C18—H18	0.9300	C47—H47	0.9300
C31—C3	1.494 (3)	C48—C49	1.360 (4)
C31—C32	1.491 (4)	C48—H48	0.9300
C31—H31A	0.9700	C49—H49	0.9300
C31—H31B	0.9700	C50—C49	1.378 (4)
C32—S1	1.698 (3)	C50—H50	0.9300
			
C32—S1—C35	91.9 (3)	H31A—C31—H31B	107.6
N2—N1—C11	122.07 (18)	C31—C32—S1	124.70 (19)
C5—N1—N2	113.51 (18)	C33—C32—S1	109.3 (5)
C5—N1—C11	124.37 (19)	C33—C32—C31	126.0 (6)
C3—N2—N1	104.44 (17)	C32—C33—C34	117.3 (9)
N41—N4—C5	130.25 (17)	C32—C33—H33	121.4
C3—N4—N41	121.59 (19)	C34—C33—H33	121.4
C3—N4—C5	108.14 (18)	C34'—C33'—H33'	122.5
C42—N41—N4	117.04 (18)	C33—C34—H34	125.6
N2—C3—N4	111.7 (2)	C35—C34—C33	108.8 (9)
N2—C3—C31	124.8 (2)	C35—C34—H34	125.6
N4—C3—C31	123.5 (2)	C33'—C34'—H34'	126.2
O1—C5—N1	128.3 (2)	C35'—C34'—C33'	107.5 (13)
O1—C5—N4	129.5 (2)	C35'—C34'—H34'	126.2
N1—C5—N4	102.10 (18)	S1—C35—H35	123.7
N1—C11—C12	112.76 (19)	C34—C35—S1	112.6 (7)
N1—C11—H11A	109.0	C34—C35—H35	123.7
N1—C11—H11B	109.0	S1'—C35'—H35'	123.1
C12—C11—H11A	109.0	C34'—C35'—S1'	113.8 (12)
C12—C11—H11B	109.0	C34'—C35'—H35'	123.1
H11A—C11—H11B	107.8	N41—C42—C43	120.4 (2)
O2—C12—C11	120.3 (2)	N41—C42—H42	119.8
O2—C12—C13	122.3 (2)	C43—C42—H42	119.8
C13—C12—C11	117.41 (19)	C42—C43—H43	119.0
C14—C13—C12	119.5 (2)	C44—C43—C42	122.0 (2)
C18—C13—C14	117.6 (2)	C44—C43—H43	119.0
C18—C13—C12	122.9 (2)	C43—C44—C45	129.4 (2)
C13—C14—H14	119.4	C43—C44—H44	115.3
C15—C14—C13	121.3 (2)	C45—C44—H44	115.3
C15—C14—H14	119.4	C46—C45—C44	122.6 (2)
C14—C15—C16	120.5 (2)	C50—C45—C44	119.5 (2)
C14—C15—H15	119.8	C50—C45—C46	117.9 (2)
C16—C15—H15	119.8	C45—C46—H46	119.7
C15—C16—C17	119.1 (3)	C47—C46—C45	120.5 (2)
C15—C16—H16	120.5	C47—C46—H46	119.7
C17—C16—H16	120.5	C46—C47—C48	120.6 (3)
C16—C17—H17	119.7	C46—C47—H47	119.7
C18—C17—C16	120.7 (3)	C48—C47—H47	119.7
C18—C17—H17	119.7	C47—C48—H48	120.2
C13—C18—H18	119.6	C49—C48—C47	119.7 (3)
C17—C18—C13	120.9 (2)	C49—C48—H48	120.2
C17—C18—H18	119.6	C48—C49—C50	120.2 (3)
C3—C31—H31B	108.6	C48—C49—H49	119.9
C3—C31—H31A	108.6	C50—C49—H49	119.9
C32—C31—C3	114.50 (18)	C45—C50—H50	119.4
C32—C31—H31A	108.6	C49—C50—C45	121.1 (3)
C32—C31—H31B	108.6	C49—C50—H50	119.4
			
N2—N1—C11—C12	−111.3 (2)	C14—C13—C18—C17	0.8 (4)
C5—N1—C11—C12	71.3 (3)	C13—C14—C15—C16	−0.8 (4)
C3—N2—N1—C5	−0.6 (2)	C14—C15—C16—C17	0.1 (4)
C3—N2—N1—C11	−178.2 (2)	C15—C16—C17—C18	1.0 (4)
N41—N4—C5—O1	−2.8 (4)	C13—C18—C17—C16	−1.5 (4)
N41—N4—C5—N1	179.14 (17)	C32—C31—C3—N2	102.4 (3)
C3—N4—C5—O1	175.3 (2)	C32—C31—C3—N4	−79.4 (3)
C3—N4—C5—N1	−2.8 (2)	C3—C31—C32—S1	101.1 (3)
C3—N4—N41—C42	169.85 (18)	C3—C31—C32—C33	−77.2 (8)
C5—N4—N41—C42	−12.3 (3)	C31—C32—S1—C35	−179.3 (4)
N41—N4—C3—N2	−179.05 (17)	C33—C32—S1—C35	−0.7 (8)
N41—N4—C3—C31	2.5 (3)	S1—C32—C33—C34	1.2 (15)
C5—N4—C3—N2	2.7 (2)	C31—C32—C33—C34	179.7 (9)
C5—N4—C3—C31	−175.77 (19)	C32—C33—C34—C35	−1.1 (17)
N4—N41—C42—C43	−178.75 (17)	C33—C34—C35—S1	0.4 (13)
N4—C3—N2—N1	−1.3 (2)	C33'—C34'—C35'—S1'	−1(2)
C31—C3—N2—N1	177.15 (19)	C34—C35—S1—C32	0.1 (7)
O1—C5—N1—N2	−176.0 (2)	N41—C42—C43—C44	−177.4 (2)
O1—C5—N1—C11	1.6 (4)	C42—C43—C44—C45	−178.5 (2)
N4—C5—N1—N2	2.1 (2)	C46—C45—C44—C43	9.7 (4)
N4—C5—N1—C11	179.67 (19)	C50—C45—C44—C43	−169.5 (2)
O2—C12—C11—N1	11.1 (3)	C44—C45—C46—C47	−178.7 (2)
C13—C12—C11—N1	−167.79 (18)	C50—C45—C46—C47	0.5 (3)
O2—C12—C13—C14	−10.8 (3)	C44—C45—C50—C49	178.9 (2)
O2—C12—C13—C18	171.6 (2)	C46—C45—C50—C49	−0.4 (3)
C11—C12—C13—C14	168.08 (19)	C45—C46—C47—C48	−0.3 (4)
C11—C12—C13—C18	−9.5 (3)	C46—C47—C48—C49	0.0 (4)
C12—C13—C14—C15	−177.4 (2)	C47—C48—C49—C50	0.1 (4)
C18—C13—C14—C15	0.3 (3)	C45—C50—C49—C48	0.1 (4)
C12—C13—C18—C17	178.4 (2)		

### Hydrogen-bond geometry (Å, °)

**Table:**

*D*—H···*A*	*D*—H	H···*A*	*D*···*A*	*D*—H···*A*
C42—H42···O1	0.93	2.23	2.916 (3)	130
C14—H14···Cg1^i^	0.93	2.91	3.501 (3)	122
C11—H11B···Cg2^ii^	0.97	2.89	3.816 (3)	159
C48—H48···Cg3^iii^	0.93	2.79	3.557 (3)	141

Symmetry codes: (i) *x*, *y*, *z*−1; (ii) *x*, −*y*−3/2, *z*−1/2; (iii) *x*, −*y*−1/2, *z*−1/2.
